# Distributed Joint Cooperative Self-Localization and Target Tracking Algorithm for Mobile Networks

**DOI:** 10.3390/s19183829

**Published:** 2019-09-04

**Authors:** Junjie Zhang, Jianhua Cui, Zhongyong Wang, Yingqiang Ding, Yujie Xia

**Affiliations:** 1 School of Physics and Electronic Information, Luoyang Normal University, Luoyang 471934, China; 2 School of Information Engineering, Zhengzhou University, Zhengzhou 450001, China

**Keywords:** mobile networks, distributed localization, variational message passing, average consensus, prediction model

## Abstract

Location information is a key issue for applications of the Internet of Things. In this paper, we focus on mobile wireless networks with moving agents and targets. The positioning process is divided into two phases based on the factor graph, i.e., a prediction phase and a joint self-location and tracking phase. In the prediction phase, we develop an adaptive prediction model by exploiting the correlation of trajectories within a short period to formulate the prediction message. In the joint positioning phase, agents calculate the cooperative messages according to variational message passing and locate themselves. Simultaneously, the average consensus algorithm is employed to realize distributed target tracking. The simulation results show that the proposed prediction model is adaptive to the random movement of nodes. The performance of the proposed joint self-location and tracking algorithm is better than the separate cooperative self-localization and tracking algorithms.

## 1. Introduction

With the development of the Internet of Things (IoT) and related new information technology, wireless sensor networks (WSNs) are expected to gradually penetrate various industries and applications [1,2,3,4], influencing all aspects of people’s lives. WSNs connect the material world with the human world. Embedded in various “things”, sensors sense the states of the “things” and transmit collected information to processing terminals via the Internet to achieve monitoring and management in real-time [5]. The information collected by sensors is meaningful only when combined with location information, which helps the administrators know what, when, and where “things” happened. Therefore, cooperative self-localization and target tracking are two key issues in location-based applications of WSNs.

In WSNs, there are a small number of anchors and thousands of agents. Anchors are equipped with Global Position System (GPS) receivers or other devices with known coordinates, while the agents use a cooperative self-localization algorithm to estimate their own positions. In addition, when a target enters the WSNs, anchors and agents work cooperatively to track and locate it. In static WSNs, anchors and agents are fixed and so their coordinates do not change with time, meaning agents only need to locate themselves once. In mobile WSNs, anchors and agents move with time. Therefore, agents need to locate themselves in a sequential manner. In contrast with static WSNs, mobile WSNs alleviate several issues, such as coverage optimization and target tracking. A plethora of localization systems are proposed in the literature. In [6], the authors provided a general overview of localization in WSNs and showed the importance of different localization approaches in modern IoT applications. In [7], the authors focused on mobile WSNs and analyzed localization algorithms in mobile WSNs.

Cooperative self-localization and target tracking are closely related because target tracking can only be carried out when the locations of agents are clear. In recent years, some researches combined self-localization with target tracking and proposed a series of joint location algorithms. In [8], a simultaneous localization and tracking algorithm was introduced to solve the problem of tracking a non-cooperative target while simultaneously localizing and calibrating the static nodes in the network. In [9], a Bayesian method based on belief propagation (BP) was proposed for the distributed sequential localization of mobile networks composed of both cooperative agents and non-cooperative targets. This provided a consistent combination of cooperative self-localization and distributed tracking. However, the motion parameters (such as velocity) and the state-transition probability density function (pdf) of each node were assumed to be available. In [10], a distributed variational filtering was presented to simultaneously localize the detecting sensors and track the target by exploiting a series of measurements generated in the sensors when the target moved through the network field.

Target tracking is divided into two categories, i.e., centralized and distributed. In centralized target tracking, the nodes that detect the target transmit their measurements related to the target to a central processing node in a hop-by-hop communication mode. Then, the central node determines the positioning of the target by uniformly processing all of the measurements. However, the communication overhead of multi-hop data transmission is very large and energy consumption is too high. In distributed target tracking, each node locates the target according to all measurements related to the target. Therefore, the key problem is how to make each node obtain all of the measurements. In [11], a scheme was proposed in which agents broadcasted their own measurements, identification numbers, and additional information, such as the ID of the target. They also received these from other nodes and updated the information. All of the agents repeated the process until each agent obtained all of the measurement information related to the target. In this way, each agent was a fusion center and could perform tracing tasks. However, much redundant information existed in the network, and the required storage space was also very large. Therefore, this method is suitable for a fully connected network with a large communication radius. Consensus algorithm [12,13,14] provides another way for each agent to communicate observations with the remaining agents engaged in the tracking. Consensus is a distributed iterative algorithm relying on communication links between neighboring nodes and can achieve entire network polymerization (such as sum, average, or maximum). This does not need a routing algorithm and is robust to changes in network topology and unreliable network environments. Therefore, consensus algorithm is widely used in distributed target tracking [15,16]. In [17], each node calculated the mean and variance of the local posterior by weighted sampling. Then, the average consensus algorithm was employed to calculate the approximation of the global posterior to complete distributed target tracking. In [18], the global likelihood function was approximated to a Gaussian distribution, and each node obtained the approximation through consensus iteration. Furthermore, a consensus algorithm, which is applicable to any exponential likelihood function, was proposed in [19].

As an implementation of approximate Bayesian inference, messaging passing algorithm is particularly well-suited to distributed calculation and has been applied to cooperative self-localization and tracking of WSNs [9,20,21]. In previous work [22], we proposed a distributed cooperative self-localization algorithm by employing variational message passing (VMP) on factor graphs for static WSNs. With regard to the non-Gaussian messages caused by the nonlinear ranging model, we approximated them to Gaussian messages by exploiting second-order Taylor expansion, which significantly reduced the computational complexity. Then, we further combined the VMP approach in [22] with BP and proposed a cooperative self-localization algorithm for mobile WSNs in [23]. In this paper, we focus on mobile networks with anchors, moving agents, and targets deployed in a two-dimensional plane. In particular, the anchors have perfect location information at all times, while the cooperative agents and the non-cooperative targets are at unknown positions and can move independently. We divide the positioning process into two phases, i.e., a prediction phase and a joint self-location and tracking phase. Firstly, an adaptive prediction model is proposed to calculate the prediction message from the previous time slot. Then, in the joint self-location and tracking phase, the agents locate themselves using the measurements from neighboring nodes (agents and anchors) and the detectable targets, and the average consensus algorithm is employed to achieve distributed positioning of the targets. The simulation results show that the proposed prediction model is more adaptive and accurate than instant prediction, linear prediction, or square prediction, and the proposed joint self-location and tracking algorithm is better than the separate cooperative self-localization and target tracking algorithms.

This paper is organized as follows. In Section 2, we first describe the system model of cooperative self-localization, and tracking. Then, the corresponding probabilistic model and factor graph are analyzed. In Section 3, the proposed distributed joint self-location and tracking algorithm are described in detail, including a prediction phase and a joint self-location and tracking phase. The performance of the proposed prediction model and joint localization algorithm are evaluated in Section 4. Finally, conclusions are drawn in Section 5.

Notations: Boldface lowercase and uppercase letters denote vectors and matrices, respectively. Superscript ( )^T^ and symbol ||·|| stand for transposition and Euclidian norm, respectively. The probability density function (pdf) of a 1D Gaussian distribution with mean μ and variance $\sigma^{2}$ is represented by $N(x;\mu ,\sigma^{2})$ while the pdf of a 2D Gaussian distribution with mean vector μ and covariance matrix V is represented by N(x;μ,V).

## 2. System Model and Factor Graph

We consider a network with anchors, moving agents, and targets deployed in a two-dimensional plane, which can be defined by a vertices set V and an edges set E, i.e., G=(V,E). Here, each vertex i∈V represents a node in the plane and each edge (i,j)∈E indicates a neighborhood structure between node *i* and node *j*. We divide set V into three parts: V=A∪M∪S, where A designates all anchors with perfect location information, M includes all agents at unknown positions, and S represents all non-cooperative targets. Moreover, the number of anchors, agents, and targets are denoted by $N_{A}$, $N_{M}$, and $N_{O}$, respectively.

Time is slotted and we assume that all agents and targets move independently. At the *k*th time slot, the position variable of node i∈V is denoted by $x_{i}^{k}≜{\left[x_{i}^{k},y_{i}^{k}\right]}^{T}$. For simplicity of illustration, the communication link between cooperative neighbors is assumed to be symmetric, which means (i,j)∈E and (j,i)∈E if $|x_{i}^{k}-x_{j}^{k}|\leq R$, where *R* is the communication radius. At the *k*th time slot, if (i,j)∈E, nodes *i* can obtain the current position of node *j* and a ranging measurement $d_{i\leftarrow j}^{k}$ with Gaussian noise $\omega_{ij}^{k}∼N\left(\omega_{ij}^{k};0,{\left(\sigma_{ij}^{k}\right)}^{2}\right)$. Consequently, the ranging measurement $d_{i\leftarrow j}^{k}$ can be formulated as

$$d_{i\leftarrow j}^{k}=||x_{i}^{k}-x_{j}^{k}||+\omega_{ij}^{k},$$

and the likelihood function of the position variables of $x_{i}^{k}$ and $x_{j}^{k}$ is a Gaussian pdf, i.e.,

$$p(d_{i\leftarrow j}^{k}\left|x_{i}^{k},x_{j}^{k})\right.∼N(d_{i\leftarrow j}^{k};||x_{i}^{k}-x_{j}^{k}||,\sigma_{ij}^{k}).$$

For convenience of description, we define $V_{o}^{k}≜A_{o}^{k}\cup M_{o}^{k}$ ($A_{o}^{k}$ for anchors and $M_{o}^{k}$ for agents) to represent all nodes who can detect target at the *k*th time slot. Similarly, let $V_{m}^{k}≜A_{m}^{k}\cup M_{m}^{k}\cup S_{m}^{k}$ denote all nodes from whom node m∈M can implement ranging measurement at the *k*th time slot. Typically, notation $S_{m}^{k}$ represents all targets that node m can detect at the *k*th time slot. Moreover, we denote the position variables of all nodes by $X^{k}≜\left\{x_{i}^{k},\forall i\in A\cup M\cup S\right\}$, the ranging measurements of node m∈M by $Z_{m}^{k}≜\left\{d_{m\leftarrow j}^{k},\forall j\in A_{m}^{k}\cup M_{m}^{k}\cup S_{m}^{k}\right\}$, and the ranging measurements of target O∈S by $Z_{o}^{k}≜\left\{d_{j\leftarrow o}^{k},\forall j\in A_{o}^{k}\cup M_{o}^{k}\right\}$, respectively. Then, let $X^{k}≜\left\{x_{i}^{k},\forall i\in V\right\}$ and $Z^{k}≜\left\{Z_{i}^{k},\forall i\in M\cup S\right\}$ represent the position variables of all nodes and ranging measurements at the *k*th time slot, respectively. Furthermore, let $X^{0:K}≜\left\{X^{k},k=0:K\right\}$ denote the position variables of all nodes from the 0th time slot to the *k*th time slot and $Z^{1:K}≜\left\{Z^{k},k=1:K\right\}$ denote the ranging measurements from the 1st time slot to the *K*th time slot, respectively.

According to Bayesian rules and the assumption that all nodes move independently, the joint a posteriori distribution of $X^{0:K}$ with given observations $Z^{0:K}$ can be formulated as

(1) $$\begin{array}{cc} p\left(X^{0:K}\left|Z^{1:K}\right.\right) & \propto p\left(X^{0}\right)\prod_{k=1}^{K}p(Z^{k}\left|X^{k}\right.)p\left(X^{k}\left|X^{k-1}\right.\right)\\ & \propto \prod_{i\in V}p(x_{i}^{0})\prod_{k=1}^{K}\prod_{m\in M}\prod_{j\in V_{m}^{k}}p(d_{m\leftarrow j}^{k}\left|x_{m}^{k},x_{j}^{k})\right.\prod_{o\in S}\prod_{j\in V_{o}^{k}}p(d_{j\leftarrow o}^{k}\left|x_{o}^{k},x_{j}^{k})\right.\prod_{i\in V}p(x_{i}^{k}|x_{i}^{k-1}) \end{array},$$

where $p(x_{i}^{0})$ is the a prior distribution of node *I*, which is assumed to be a Gaussian pdf with mean $\mu_{i}^{0}$ and covariance matrix $V_{i}^{0}≜\sigma_{i0}^{2}I_{2\times 2}$, $p(d_{i\leftarrow j}^{k}\left|x_{i}^{k},x_{j}^{k})\right.$ is the likelihood function of node *i* and node *j*, and $p(x_{i}^{k}|x_{i}^{k-1})$ is the probabilistic state-transition function.

**Figure 1:**
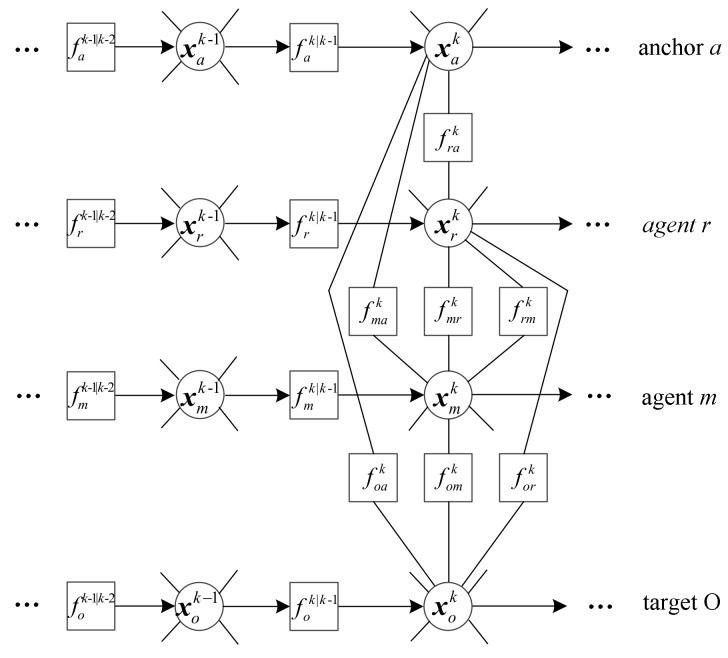
The factor graph corresponding to the factorization in (1).

For simplicity, we define $f_{i}^{k|k-1}≜p\left(x_{i}^{k}|x_{i}^{k-1}\right)$ and $f_{ij}^{k}≜p(d_{i\leftarrow j}^{k}\left|x_{i}^{k},x_{j}^{k})\right.$. Based on the factorization in (1), the joint a posteriori distribution of $p(X^{0:K}\left|Z^{0:K}\right.)$ can be represented by a factor graph, as shown in Figure 1. In the factor graph, each variable node represents the position variable $x_{i}^{k}$ of node *i* and is depicted by a circle, while each factor node represents a local function $f_{i}^{k|k-1}$ or $f_{ij}^{k}$ and is drawn by a square. Moreover, if a variable is an argument of a local function, it is connected to the factor node through an edge.

Generally speaking, factor graphs are undirected digraphs, which means the edge between a factor node and a variable node is undirected. However, in Figure 1, messages only flow forward in time regarding the spatiotemporal constraints of the network. We do not calculate the messages from the present to the past because network connectivity may change and the states of the nodes may be outdated. Furthermore, the variable node of an anchor’s position ignores the messages from its neighbors as anchors’ positions are known.

## 3. Distributed Joint Self-Location and Tracking Algorithm

As shown in Figure 1, for the cooperative agent m∈M and the non-cooperative target O∈S, the beliefs of $x_{m}^{k}$ and $x_{O}^{k}$, denoted by $b(x_{m}^{k})$ and $b(x_{o}^{k})$, consist of two kinds of message: Prediction messages from the (*k*−1)th time slot and cooperative messages from the neighbors. Based on message passing rules, for node i∈M∪S, the belief $b(x_{i}^{k})$ can be described as

(2) $$b\left(x_{i}^{k}\right)=\frac{1}{Z}m_{f_{i}^{k|k-1}\to x_{i}^{k}}\left(x_{i}^{k}\right)\prod_{j\in V_{i}^{k}}m_{f_{ij}^{k}\to x_{i}^{k}}\left(x_{i}^{k}\right),$$

where *Z* is the normalization constant and $m_{f_{i}^{k|k-1}\to x_{i}^{k}}\left(x_{i}^{k}\right)$ and $m_{f_{ij}^{k}\to x_{i}^{k}}\left(x_{i}^{k}\right)$ represent messages from factor node and factor nodes $f_{ij}^{k}$ ($\forall j\in V_{i}^{k}$) to variable node $x_{i}^{k}$, respectively. Therefore, agent m∈M determines $b(x_{m}^{k})$ and $b(x_{o}^{k})$($m\in V_{o}^{k}$) by two phases, i.e., a prediction phase and a joint self-location and tracking phase.

### 3.1. Adaptive Prediction Model and Prediction Message Calculating

In (1), the probabilistic state-transition function $p(x_{i}^{k}|x_{i}^{k-1})$ of node *i* is related to the position prediction model. In this paper, according to the inertia of the motion, we propose an adaptive prediction model by exploiting the correlation of trajectories of node *i* within a short period.

We denote the trajectory of node *i* from the (*k−N*)th time slot to the (*k*−1)th time slot as $T_{i}(k-1,N)≜{\left({\left({\hat{x}}_{i}^{k-1}\right)}^{T},{\left({\hat{x}}_{i}^{k-2}\right)}^{T},\cdots ,{\left({\hat{x}}_{i}^{k-N}\right)}^{T}\right)}^{T}$, where ${\hat{x}}_{i}^{k-N}≜{\left[{\hat{x}}_{i}^{k-N},{\hat{y}}_{i}^{k-N}\right]}^{T}$, …,${\hat{x}}_{i}^{k-1}≜{\left[{\hat{x}}_{i}^{k-1},{\hat{y}}_{i}^{k-1}\right]}^{T}$ are the estimated positions of node *i* at the (*k−N*)th, …, (*k*−1)th time slot. Similarly, the trajectory of node *i* from the (*k*−*N* + 1)th time slot to the *k*th time slot can be described as $T_{i}(k,N)≜{\left({\left({\hat{x}}_{i}^{k}\right)}^{T},{\left({\hat{x}}_{i}^{k-1}\right)}^{T},\cdots ,{\left({\hat{x}}_{i}^{k-N+1}\right)}^{T}\right)}^{T}$. Then, we formulate the prediction model of node *i* as

(3) $$T_{i}(k,N)=P^{i}\cdot T_{i}(k-1,N)\;,$$

where $P^{i}$ is the prediction matrix of node *i* and its dimension is 2*N* × 2*N*. Obviously, there are only two different elements between $T_{i}(k-1,N)$ and $T_{i}(k,N)$, i.e., ${\hat{x}}_{i}^{k}$ and ${\hat{x}}_{i}^{k-N}$. Therefore, the elements from the 3rd to the 2*N*th rows are

(4) $$p_{r,s}^{i}=\left\{\begin{array}{ll} 0, & r\geq 3\;\mathrm{and}\;r\neq s+2\\ 1, & r\geq 3\;\mathrm{and}\;r=s+2 \end{array}\right..$$

According to the inertia of the motion of node *i*, we assume that the prediction matrix $P^{i}$ is unchanged in a short period. Based on the prediction model in (4), we have

(5) $$T_{i}(k-1,N)=P^{i}\cdot T_{i}(k-2,N)\;.$$

Consequently, it can be formulated as

(6) $$T_{i}(k,N)={\left(P^{i}\right)}^{n}\cdot T_{i}(k-n,N).$$

Based on Equations (3) and (5), we have

(7) $$T_{i}(k,N)-T_{i}(k-1,N)=P^{i}\cdot [T_{i}(k-1,N)-T_{i}(k-2,N)]\;\;\;.$$

Obviously, translational motion will not change the prediction matrix $P^{i}$. Therefore, it can be expressed as

(8) $$T_{i}(k,N)-H=P^{i}\cdot [T_{i}(k-1,N)-H]\;,$$

and H satisfies the constraint as follows

(9) $$H={\left(P^{i}\right)}^{n}\cdot H,$$

where $H≜{\left[a,b,\ldots ,a,b\right]}_{2N\times 1}^{T}$ and its two base vectors are $h_{1}≜{\left[1\;0\;1\;0\ldots 1\;0\right]}^{T}$ and $h_{2}≜{\left[0\;1\;0\;1\ldots 0\;1\right]}^{T}$. Then we have $h_{1}=P^{i}\cdot h_{1}$ and $h_{2}=P^{i}\cdot h_{2}$.

Assume that each node stores *M* (M>N) estimated positions before the *k*th time slot, which constitute M−N+1 trajectories, i.e., $T_{i}(k-1,N)$, $T_{i}(k-2,N)$,…, $T_{i}(k-M+N-1,N)$. Based on the prediction model in Equation (3), we obtain an equation set which can be expressed as

(10) $$\left\{\begin{array}{c} T_{i}(k-1,N)=P^{i}\times T_{i}(k-2,N)\\ T_{i}(k-2,N)=P^{i}\times T_{i}(k-3,N)\\ \vdots \\ T_{i}(k-M+N,N)=P^{i}\times T_{i}(k-M+N-1,N) \end{array}\right..$$

According to Equations (4) and (10), we have

(11) $$\left\{ \begin{array}{l} \left(\begin{array}{cc} 1 & 0 \end{array}\right)=h_{1}^{T}\times \left(\begin{array}{cc} {\left(p_{1,*}^{i}\right)}^{T} & {\left(p_{2,*}^{i}\right)}^{T} \end{array}\right)\\ \left(\begin{array}{cc} 0 & 1 \end{array}\right)=h_{2}^{T}\times \left(\begin{array}{cc} {\left(p_{1,*}^{i}\right)}^{T} & {\left(p_{2,*}^{i}\right)}^{T} \end{array}\right) \end{array},\right.$$

where $p_{1,*}^{i}$ and $p_{2,*}^{i}$ are the first and second rows of $P^{i}$. For convenience of description, we define

$$B_{i,M-N}≜\left[\begin{array}{cc} \begin{array}{c} {\hat{x}}_{i}^{k-1}\\ \begin{array}{c} {\hat{x}}_{i}^{k-2}\\ \vdots \end{array}\\ {\hat{x}}_{i}^{k-M+N}\\ \begin{array}{c} 1\\ 0 \end{array} \end{array} & \begin{array}{c} {\hat{y}}_{i}^{k-1}\\ \begin{array}{c} {\hat{y}}_{i}^{k-2}\\ \vdots \end{array}\\ {\hat{y}}_{i}^{k-M+N}\\ \begin{array}{c} 0\\ 1 \end{array} \end{array} \end{array}\right],\;T_{i,M-N}≜\left[\begin{array}{c} {\left(T_{i}(k-2,N)\right)}^{T}\\ \begin{array}{c} {\left(T_{i}(k-3,N)\right)}^{T}\\ \vdots \end{array}\\ {\left(T_{i}(k-M+N-1,N)\right)}^{T}\\ \begin{array}{c} h_{1}^{T}\\ h_{2}^{T} \end{array} \end{array}\right].$$

Then, according to Equations (4), (10), and (11), we have

(12) $$B_{i,M-N}=T_{i,M-N}\cdot \left(\begin{array}{cc} {\left(p_{1,*}^{i}\right)}^{T} & {\left(p_{2,*}^{i}\right)}^{T} \end{array}\right).$$

Consequently, $p_{1,*}^{i}$ and $p_{2,*}^{i}$ can be determined based on the least square method, i.e.,

(13) $$\left(\begin{array}{l} p_{1,*}^{i}\\ p_{2,*}^{i} \end{array}\right)=B_{i,M-N}^{T}\cdot T_{i,M-N}\cdot {\left[{\left(T_{i,M-N}^{T}\cdot T_{i,M-N}\right)}^{-1}\right]}^{T}.$$

Finally, the prediction matrix ***P**^i^* is given by Equations (4) and (13). Let ${\tilde{x}}_{i}^{k}≜{\left[{\tilde{x}}_{i}^{k},{\tilde{y}}_{i}^{k}\right]}^{T}$ denote the predicted position of node *i*. According to the prediction model in Equation (3), ${\tilde{x}}_{i}^{k}$ can be given by

(14) $${\tilde{x}}_{i}^{k}=\left(\begin{array}{l} p_{1,*}^{i}\\ p_{2,*}^{i} \end{array}\right)\cdot T_{i}(k-1,N)$$

Then, we use the predicted position ${\tilde{x}}_{i}^{k}$ to formulate the prediction message $m_{f_{i}^{k|k-1}\to x_{i}^{k}}(x_{i}^{k})$ as

(15) $$m_{f_{i}^{k|k-1}\to x_{i}^{k}}\left(x_{i}^{k}\right)=N\left(x_{i}^{k};{\tilde{x}}_{i}^{k},{\tilde{V}}_{i}^{k}\right),$$

where the covariance matrixes are ${\tilde{V}}_{i}^{k}={\tilde{\sigma }}_{i,k}^{2}I_{2\times 2}$ and ${\tilde{\sigma }}_{i,k}^{2}=||{\hat{x}}_{i}^{k-1}-{\tilde{x}}_{i}^{k-1}|{|}^{2}$.

### 3.2. Joint Self-Location and Tracking Phase

After calculating the prediction message based on the adaptive prediction model, agent m∈M locates itself using ranging measurements, not only from its neighbor anchors and agents $j\in A_{i}^{k}\cup M_{i}^{k}$, but also from targets $O\in S_{i}^{k}$ that it can detect. Meanwhile, agents and anchors that can detect target *O* share information with each other based on consensus iteration and determine the position of the target *O*. Compared with the separate cooperative self-localization and algorithms, agents use the ranging measurements from targets to improve the self-location accuracy, which promotes tracking performance in turn.

#### 3.2.1. Self-Location of Agents Based on Variational Message Passing

At the *k*th time slot, agent m∈M obtains its prediction message $m_{f_{m}^{k|k-1}\to x_{m}^{k}}\left(x_{m}^{k}\right)=N\left(x_{m}^{k};{\tilde{x}}_{m}^{k},{\tilde{V}}_{m}^{k}\right)$ based on the adaptive prediction model described in Section 3. Then, according to VMP update rules, cooperative messages from its neighbors are expressed as follows:

(16) $$\begin{array}{cc} m_{f_{ma}^{k}\to x_{m}^{k}}\left(x_{m}^{k}\right) & ≜\exp\left\{\int m_{x_{a}^{k}\to f_{ma}^{k}}\left(x_{a}^{k}\right)\ln f_{ma}^{k}\left(x_{a}^{k},x_{m}^{k}\right)dx_{a}^{k}\right\}\\ & =N\left(d_{m\leftarrow a}^{k};∥x_{m}^{k}-\mu_{a}^{k}∥,{\left(\sigma_{ma}^{k}\right)}^{2}\right), \end{array}$$

(17) $$\begin{array}{cc} m_{f_{mj}^{k}\to x_{m}^{k}}\left(x_{m}^{k}\right) & =\exp\left\{\int m_{x_{j}^{k}\to f_{mj}^{k}}\ln p\left(d_{m\leftarrow j}^{k}|x_{m}^{k},x_{j}^{k}\right)dx_{j}^{k}\right\}\\ & =\exp\left\{\int b\left(x_{j}^{k}\right)\ln N\left(d_{m\leftarrow j}^{k};∥x_{m}^{k}-x_{j}^{k}∥,{\left(\sigma_{mj}^{k}\right)}^{2}\right)dx_{j}^{k}\right\}. \end{array}$$

In (16), $\mu_{a}^{k}$ is the position of anchor a∈A at the *k*th time slot. Consequently, the belief $b(x_{m}^{k})$ in Equation (2) can be rewritten as

(18) $$\begin{array}{cc} b\left(x_{m}^{k}\right) & =\frac{1}{Z}N\left(x_{m}^{k};{\tilde{x}}_{m}^{k},{\tilde{V}}_{m}^{k}\right)\times \prod_{a\in A_{m}^{k}}N\left(d_{m\leftarrow a}^{k};∥x_{m}^{k}-\mu_{a}^{k}∥,{\left(\sigma_{ma}^{k}\right)}^{2}\right)\\ & \times \prod_{j\in M_{m}^{k}\cup S_{m}^{k}}\exp\left\{\int b\left(x_{j}^{k}\right)\ln N\left(d_{m\leftarrow j}^{k};∥x_{m}^{k}-x_{j}^{k}∥,{\left(\sigma_{mj}^{k}\right)}^{2}\right)dx_{j}^{k}\right\}. \end{array}$$

Considering the communication overhead, the messages passed on in Figure 1 are restricted to Gaussian distribution. However, $b(x_{m}^{k})$, given by Equation (18), is non-Gaussian because of the nonlinear ranging model. In [20], we propose a low-complexity approximation method based on second-order Taylor expansion. In this way, the belief $b(x_{m}^{k})$ is approximated into a Gaussian function denoted by $\hat{b}(x_{m}^{k})∼N\left(x_{m}^{k};{\hat{\mu }}_{m}^{k},{\hat{V}}_{m}^{k}\right)\;$, where $\mu_{i}^{k}$ and $V_{i}^{k}$ are as follows:

(19) $$V_{m}^{k}≜{\left\{{\left({\tilde{V}}_{m}^{k}\right)}^{-1}+\sum_{a\in A_{m}^{k}}\frac{I_{2\times 2}-d_{m\leftarrow a}^{k}\nabla_{F_{ma}}^{2}}{{\left(\sigma_{ma}^{k}\right)}^{2}}+\sum_{j\in M_{i}^{k}}\frac{1}{{\left(\sigma_{mj}^{k}\right)}^{2}}[I_{2\times 2}-d_{m\leftarrow j}^{k}\frac{\partial^{2}F_{mj}}{\partial {\left(x_{m}^{k}\right)}^{2}}]\right\}}^{-1},$$

(20) $$\mu_{m}^{k}≜{\hat{V}}_{m}^{k}\left\{{\left({\tilde{V}}_{m}^{k}\right)}^{-1}{\tilde{x}}_{m}^{k}\right.\;+\sum_{a\in A_{m}^{k}}\frac{\mu_{a}^{k}-d_{i\leftarrow a}^{k}(\nabla_{F_{ma}}-\nabla_{F_{ma}}^{2}\mu_{m}^{k*})}{{\left(\sigma_{ma}^{k}\right)}^{2}}\;+\left.\sum_{j\in M_{m}^{k}\cup S_{m}^{k}}\frac{1}{{\left(\sigma_{mj}^{k}\right)}^{2}}[\mu_{j}^{k*}+d_{m\leftarrow j}^{k}(\frac{\partial F_{mj}}{\partial x_{m}^{k}}-\frac{\partial^{2}F_{mj}}{\partial {\left(x_{m}^{k}\right)}^{2}}\mu_{m}^{k*})]\right\}.$$

In Equations (19) and (20), the notations $\mu_{m}^{k*}$ and $\mu_{j}^{k*}$ represent the estimated positions of nodes *m* and *j* in the latest iteration, respectively, notations $\nabla_{F_{ma}}$ and $\nabla_{F_{ma}}^{2}$ are the first-order gradient and the Hessian matrix of $F_{ma}^{k}≜\left|\right|x_{m}^{k}-\mu_{a}^{k}\left|\right|$ at $\mu_{m}^{k*}$, respectively, and the notations $\frac{\partial F_{mj}}{\partial x_{m}^{k}}$ and $\frac{\partial^{2}F_{mj}}{\partial {\left(x_{m}^{k}\right)}^{2}}$ are the first-order partial derivatives of $F_{mj}^{k}≜||x_{m}^{k}-x_{j}^{k}||$ and $\frac{1}{\partial x_{m}^{k}}(\frac{\partial F_{mj}}{\partial x_{m}^{k}})$ at ($\mu_{m}^{k*}$,$\mu_{j}^{k*}$), respectively (please see [22] for detailed derivation). Consequently, agent i∈M obtains its location based on maximum a posteriori (MAP), i.e., ${\hat{x}}_{i}^{k}=\mu_{i}^{k}$.

#### 3.2.2. Target Tracking Based on Average Consensus

At *k*th time slot, node $i\in S_{o}^{k}$, which can detect target O∈S, computes the prediction message $m_{f_{o}^{k|k-1}\to x_{o}^{k}}\left(x_{o}^{k}\right)=N\left(x_{o}^{k};{\tilde{x}}_{o}^{k},{\tilde{V}}_{o}^{k}\right)$ and the local message $m_{f_{oi}^{k}\to x_{o}^{k}}\left(x_{o}^{k}\right)$. Then, $m_{f_{oi}^{k}\to x_{o}^{k}}\left(x_{o}^{k}\right)$ is approximated into Gaussian function $N(x_{o}^{k};\mu_{oi}^{k},V_{oi}^{k})$ using the approximation method in [22]. Therefore, the Gaussian approximation of $b(x_{o}^{k})$ can be expressed as

(21) $$\hat{b}\left(x_{o}^{k}\right)=\frac{1}{Z}N\left(x_{o}^{k};{\tilde{x}}_{o}^{k},{\tilde{V}}_{o}^{k}\right)\times \prod_{i\in V_{o}^{k}}N(x_{o}^{k};\mu_{oi}^{k},V_{oi}^{k}).$$

The mean vector $\mu_{o}^{k}$ and the covariance matrix $V_{o}^{k}$ are given by ${\left(V_{o}^{k}\right)}^{-1}={\left({\tilde{V}}_{o}^{k}\right)}^{-1}+\sum_{i\in V_{o}^{k}}{\left(V_{oi}^{k}\right)}^{-1}$ and $\mu_{o}^{k}=V_{o}^{k}\left({\left({\tilde{V}}_{i}^{k}\right)}^{-1}\mu_{i}^{k}+\sum_{i\in V_{o}^{k}}{\left(V_{oi}^{k}\right)}^{-1}\mu_{oi}^{k}\right)$, respectively. Each node $i\in S_{o}^{k}$ can calculate ${\tilde{V}}_{o}^{k}$ and $V_{oi}^{k}$ locally. However, node $i\in S_{o}^{k}$ cannot determine $\hat{b}(x_{o}^{k})$ unless all other neighbors of target O share their local information with it.

For convenience of discussion, let $W_{o}^{k}≜{\left(V_{o}^{k}\right)}^{-1}$, ${\tilde{W}}_{o}^{k}≜{\left({\tilde{V}}_{o}^{k}\right)}^{-1}$ and $W_{oi}^{k}≜{\left(V_{oi}^{k}\right)}^{-1}$; therefore, we have

(22) $$W_{o}^{k}={\tilde{W}}_{o}^{k}+\sum_{i\in V_{o}^{k}}W_{oi}^{k},$$

(23) $$W_{o}^{k}\mu_{o}^{k}={\tilde{W}}_{o}^{k}{\tilde{x}}_{o}^{k}+\sum_{i\in V_{o}^{k}}W_{oi}^{k}\mu_{oi}^{k}.$$

In order to locate the target in a distributed manner, we use the average consensus based on Metropolis weights proposed in [13] to diffuse information across the network. During the process, each node updates its data with a weighted average of its neighbors’ data, which converges to the global average. Let *t* denote the iteration index and $N_{i}^{k}$ represent the number of neighbors of node *i*, respectively. The consensus iteration process is as follows.

Step 1: Each node $i\in V_{o}^{k}$ uses local information to initialize the global average $W_{i}^{k,(0)}$ and $W_{i}^{k,(0)}\mu_{i}^{k,(0)}$ as

$$W_{i}^{k,(0)}=W_{oi}^{k},W_{i}^{k,(0)}\mu_{i}^{k,(0)}=W_{oi}^{k}\mu_{oi}^{k},$$

while each node $i\notin V_{o}^{k}$ initializes $W_{i}^{k,(0)}$ and $W_{i}^{k,(0)}\mu_{i}^{k,(0)}$ as

$$W_{i}^{k,(0)}=0,W_{i}^{k,(0)}\mu_{i}^{k,(0)}=0.$$

Step 2: Each node computes the Metropolis weight matrix, which is defined as

(24) $$\xi_{i,j}^{\left(t\right)}=\left\{\begin{array}{cc} \frac{1}{1+\max\{N_{i}^{k},N_{j}^{k}\}} & \mathrm{if}\;j\in V_{i}^{k},\\ 1-\sum_{l\in V_{i}^{k}}\xi_{i,l}^{\left(t\right)} & \mathrm{if}\;i=j,\\ 0 & \mathrm{otherwise}. \end{array}\right.$$

Step 3: Each node broadcasts its global average computed in the latest iteration and collects the information broadcasted by its neighbors. Then, each node updates the global average $W_{i}^{k,(t+1)}$ and $W_{i}^{k,(t+1)}\mu_{i}^{k,(t+1)}$ by

(25) $$W_{i}^{k,(t+1)}=\xi_{i,i}^{\left(t\right)}W_{i}^{k,(t)}+\sum_{j\in V_{i}^{k}}\xi_{i,j}^{\left(t\right)}W_{j}^{k,(t)},$$

(26) $$W_{i}^{k,(t+1)}\mu_{i}^{k,(t+1)}=\xi_{i,i}^{\left(t\right)}W_{i}^{k,(t)}\mu_{i}^{k,(t)}+\sum_{j\in V_{i}^{k}}\xi_{i,j}^{\left(t\right)}W_{j}^{k,(t)}\mu_{j}^{k,(t)}.$$

Step 4: If $t=t_{\max}$ (where $t_{\max}$ is the maximum number of iteration), the consensus process ends. Otherwise, go to Step 2.

After sufficient consensus iterations, all nodes obtain the global average of $\sum_{i\in V_{o}^{k}}W_{oi}^{k}$ and $\sum_{i\in V_{o}^{k}}W_{oi}^{k}\mu_{oi}^{k}$. Therefore, each node $i\in V_{o}^{k}$ can compute $\sum_{i\in V_{o}^{k}}W_{oi}^{k}$ and $\sum_{i\in V_{o}^{k}}W_{oi}^{k}\;\mu_{oi}^{k}$ by

(27) $$\sum_{i\in V_{o}^{k}}W_{oi}^{k}=N_{o}^{k}\times W_{i}^{k,(t_{\max})},$$

(28) $$\sum_{i\in V_{o}^{k}}W_{oi}^{k}\mu_{oi}^{k}=N_{o}^{k}\times W_{i}^{k,(t_{\max})}\mu_{i}^{k,(t_{\max})}.$$

Submitting Equations (27) and (28) to Equations (22) and (23), each node $i\in V_{o}^{k}$ has

(29) $$W_{o}^{k}={\tilde{W}}_{o}^{k}+N_{o}^{k}\times W_{i}^{k,(t_{\max})},$$

(30) $$W_{o}^{k}\mu_{o}^{k}={\tilde{W}}_{o}^{k}{\tilde{x}}_{o}^{k}+N_{o}^{k}\times W_{i}^{k,(t_{\max})}\mu_{i}^{k,(t_{\max})}.$$

Therefore, each node $i\in V_{o}^{k}$ can determine the position of target O based on MAP, i.e., ${\hat{x}}_{o}^{k}=\mu_{o}^{k}$.

### 3.3. Implementation Scheme of the Joint Self-Location and Tracking Algorithm

The proposed distributed joint self-location and tracking algorithm based on VMP and average consensus is stated as follows.


**Algorithm 1: Distributed joint self-location and tracking algorithm**
1for $k=1:k_{\max}$(*k* is the time slot index)2Each agent m∈M updates *M* historical position information of itself and the targets before the *k*th time slot.3Each agent m∈M calculates its prediction matrix $P^{m}$ using Equation (4) and Equation (13), then predicts its position ${\tilde{x}}_{m}^{k}$ using Equations (14) and obtains its prediction message $m_{f_{m}^{k|k-1}\to x_{m}^{k}}(x_{m}^{k})$ using Equation (15).4Each node $i\in S_{o}^{k}$ predicts the position of target O using Equation (4), Equation (13), and Equation (14), i.e., ${\tilde{x}}_{o}^{k}$. Then, the prediction message $m_{f_{o}^{k|k-1}\to x_{o}^{k}}(x_{o}^{k})$ is calculated using Equation (15).5Initialization: Each agent m∈M initializes its position as $x_{m}^{k}={\tilde{x}}_{m}^{k}$ and initializes the position of the target that it detects as $x_{o}^{k}={\tilde{x}}_{o}^{k}$.6for $q=1:q_{\max}$(where *q* is the iteration index)7
(1)Each agent m∈M collects ranging measurements from its neighbors and calculates cooperative messages $m_{f_{ma}^{k}\to x_{m}^{k}}^{\left(q\right)}\left(x_{m}^{k}\right)$($\forall a\in A_{m}^{k}$) from neighbor anchors using Equation (16) and $m_{f_{mj}^{k}\to x_{m}^{k}}^{\left(q\right)}\left(x_{m}^{k}\right)$($\forall j\in M_{m}^{k}\cup S_{m}^{k}$) from neighbor agents and targets using Equation (17), respectively. Then, each agent m∈M calculates $\mu_{m}^{k,(q)}$ and $V_{m}^{k,(q)}$ of ${\hat{b}}^{\left(p\right)}\left(x_{m}^{k}\right)$ using Equations (19) and (20), respectively.
8
(2)Each node $i\in S_{o}^{k}$ receives the local message $m_{f_{oi}^{k}\to x_{o}^{k}}^{\left(q\right)}(x_{o}^{k})$ and approximates it into Gaussian function $N(x_{o}^{k};\mu_{oi}^{k,(q)},V_{oi}^{k,(q)})$ based on the Taylor expansion approximation method in [20]. Then the global average is calculated based on average consensus iteration from Step 1 to Step 4, which are depicted in Section 3.2.2. Then, each node $i\in S_{o}^{k}$ computes $W_{o}^{k,(q)}$ and $W_{o}^{k,(q)}\mu_{o}^{k,(q)}$ using Equations (27)–(30).
9
(3)Each agent m∈M broadcasts the mean $\mu_{m}^{k,(q)}$ and the covariance matrix $V_{m}^{k,(q)}$ of ${\hat{b}}^{\left(p\right)}(x_{m}^{k})$. Each node $i\in S_{o}^{k}$ broadcasts $W_{o}^{k,(q)}\mu_{o}^{k,(q)}$ and $W_{o}^{k,(q)}$. At the same time, they collect the information broadcasted by its neighbors.
10end for *q*11Each agent m∈M determines its position based on MAP and broadcasts $\mu_{m}^{k}$ and $V_{m}^{k}$. Each node $i\in S_{o}^{k}$ determines the position of target O based on based on MAP and broadcast $W_{o}^{k}$ and $W_{o}^{k}\mu_{o}^{k}$.12end for *k*

## 4. Stimulation Results and Analysis

In this section, we evaluate the performance of the proposed adaptive prediction model and the distributed joint self-location and tracking algorithm (denoted by JSLT).

### 4.1. Stimulation Results and Performance Analysis of the Proposed Adaptive Prediction Model

**Figure 2:**
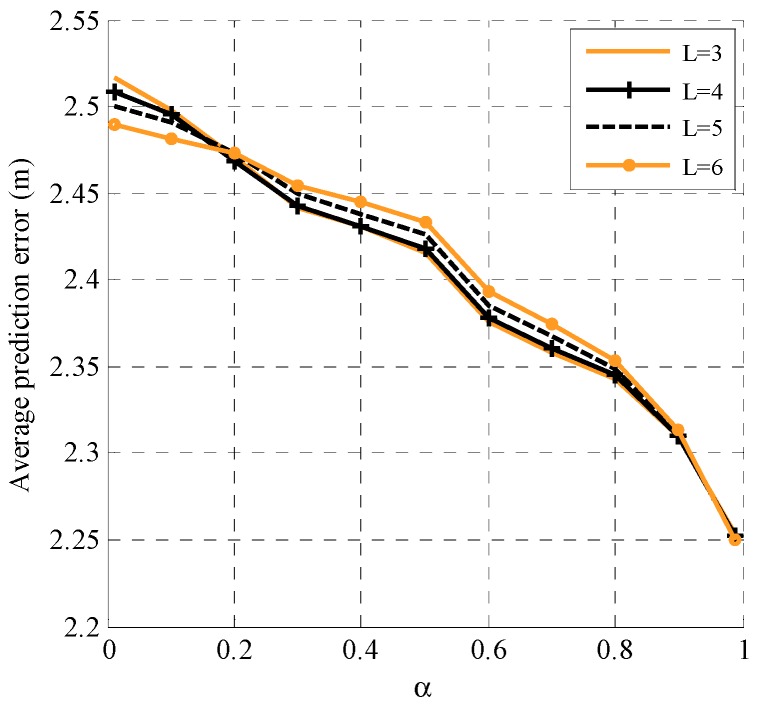
The performance of the proposed adaptive prediction model with different numbers of historical positions and an adjustment parameter α.

**Figure 3:**
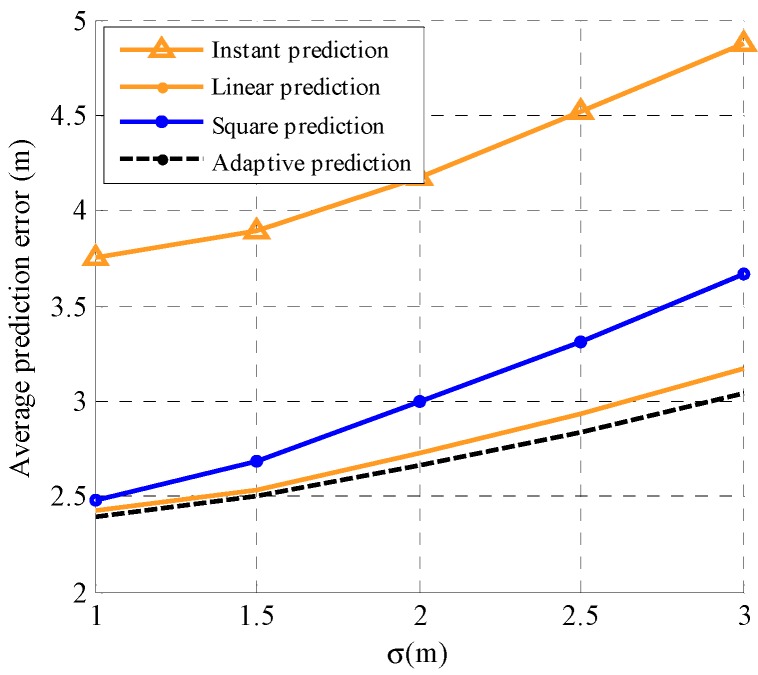
The performance comparison of the four prediction models with different standard deviations of the random variables.

**Figure 4:**
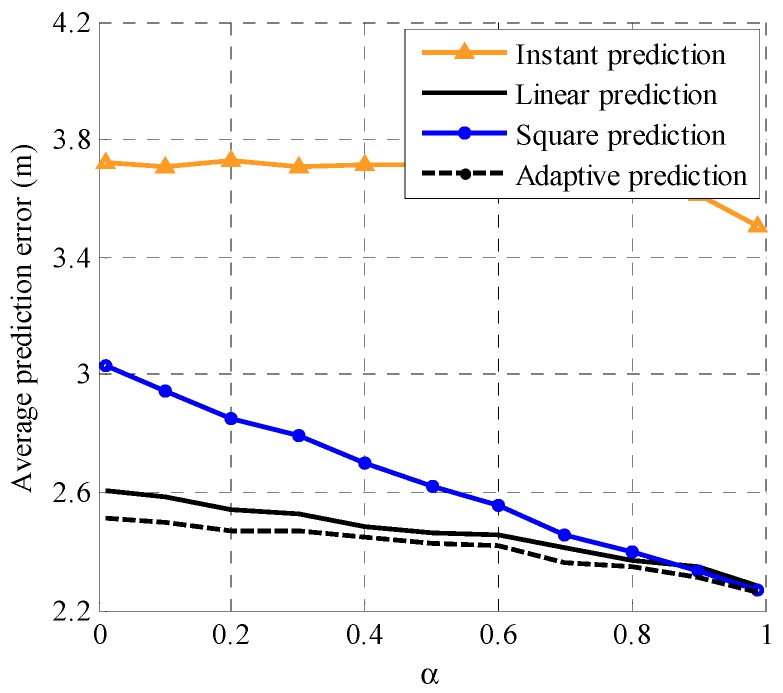
The performance comparison of the four prediction models with different adjustment parameter α values.

In order to assess the performance of the proposed adaptive prediction model, we compare it with linear prediction and square prediction. We consider a 50 m × 50 m plane with 10 moving nodes. The prediction error is defined as the distance between the true position and the prediction position. The average prediction errors in Figure 2, Figure 3 and Figure 4 are obtained by averaging over 10,000 realizations, with each realization continuing 20 time slots.

We employ the Gauss–Markov (GM) mobility model [24] to simulate the motion of nodes. In the GM model, the velocity and direction of nodes are time-dependent Gauss–Markov processes, which means the velocity and direction at the *k*th time slot is related to that at the (*k*−1)th time slot. Let $d_{i}^{k}$ and $\theta_{i}^{k}$ denote the velocity and direction of node *i* from the *k*th time slot to the (*k*−1)th time slot, respectively, then we have

(31) $$\left\{\begin{array}{l} d_{i}^{k}=\alpha d_{i}^{k-1}+(1-\alpha ){\bar{d}}_{i}+\sqrt{1-\alpha^{2}}\omega_{d}\\ \theta_{i}^{k}=\alpha \theta_{i}^{k-1}+(1-\alpha ){\bar{\theta }}_{i}+\sqrt{1-\alpha^{2}}\omega_{\theta } \end{array}\right.,$$

where constants ${\bar{d}}_{i}$ and ${\bar{\theta }}_{i}$ are the velocity and direction of node *i* when k→∞, $\omega_{d}$ and $\omega_{\theta }$ are Gaussian random variables independent of ${\bar{d}}_{i}$ and ${\bar{\theta }}_{i}$, and α∈[0,1] is an adjustment parameter to control the randomness of motion. When α increases, the impact of $d_{i}^{k-1}$ and $\theta_{i}^{k-1}$ rises, while the impact of $\omega_{d}$ and $\omega_{\theta }$ declines. As discussed above, the Gauss–Markov mobility model not only reflects the relationship between the current moment and a historical moment, but also reflects the randomness of motion. Therefore, it is very suitable for the simulation of high random motion, such as the travel of birds and the diffusion of gas.

The performance of the proposed adaptive prediction model with different numbers of historical estimated positions and α is illustrated in Figure 2. Simulation configurations are as follows. For the GM mobility model shown in Equation (31), we set ${\bar{d}}_{i}=5\;m/s$ and ${\bar{\theta }}_{i}=60^{{}^{\circ}}$. For the prediction using Equation (13), the parameters are N=1 and M=3/4/5/6. As we know, the prediction algorithm is based on the historical estimated positions. In initialization, we use the true positions along with Gaussian noise (mean = 0 and variance = 4 m^2^) as the estimated positions. As shown in Figure 2, there is little difference in the average prediction error using different historical positions with different α. Comparatively speaking, when the value of α is smaller, more historical positions are used and the more accurate the obtained prediction is; when the value of α is bigger, more historical positions are used and a more accurate prediction is achieved. This is because a smaller α value indicates a greater randomness of motion, therefore the impact of the (*k*−1)th time slot on the *k*th time slot is less. More historical positions can reflect the trend of the movement. However, a bigger α value leads to a stronger correlation between the *k*th time slot and the (*k*−1)th time slot. In this situation, the contribution of the earlier time slots to the current moment is small, and using too much historical information even influences the accuracy of prediction. Moreover, greater the number of historical positions used, the more information the nodes store and the greater the energy consumption. In subsequent simulations, we use three historical positions, meaning each node stores three historical positions.

To further evaluate the performance of the proposed adaptive prediction model, we compare the proposed model with linear prediction, square prediction, and instant prediction, which use the movements in *x* axis and *y* axis at the (*k*−1)th time slot to predict the position at the *k*th time slot. Simulation configurations are α=0.7, ${\bar{d}}_{i}=3\;m/s$, ${\bar{\theta }}_{i}=60^{{}^{\circ}}$, N=1, and M=3. The performance comparison of the four prediction models with different standard deviations $\sigma_{\omega_{d}}=\sigma_{\omega_{\theta }}=\sigma$ of the random variables $\omega_{d}$ and $\omega_{\theta }$ is shown in Figure 3. It is observed that the average prediction error of the four prediction models increases with an increase in σ. Specifically, the performance of instant prediction is the worst and the performance of the proposed adaptive prediction is the best. Moreover, the advantage of the proposed adaptive prediction model is more obvious when σ is bigger. Figure 4 illustrates the performance of the four prediction models with different adjustment parameter α values. Simulation configurations are α=0.7, ${\bar{d}}_{i}=3\;m/s$, ${\bar{\theta }}_{i}=60^{{}^{\circ}}$, σ=1 m and M=3. It can be observed that the average prediction error of the proposed adaptive prediction model is smaller than the other three prediction models and the difference is bigger with increasing α. In fact, from the Gauss–Markov mobility model in Equation (31), we see that the randomness of motion rises with the decline of α and the increase in σ. Therefore, the proposed adaptive prediction model better adapts to different motion patterns than the other three prediction models.

### 4.2. Stimulation Results and Performance Analysis of the Proposed Distributed Joint Self-Location and Tracking Algorithm

**Figure 5:**
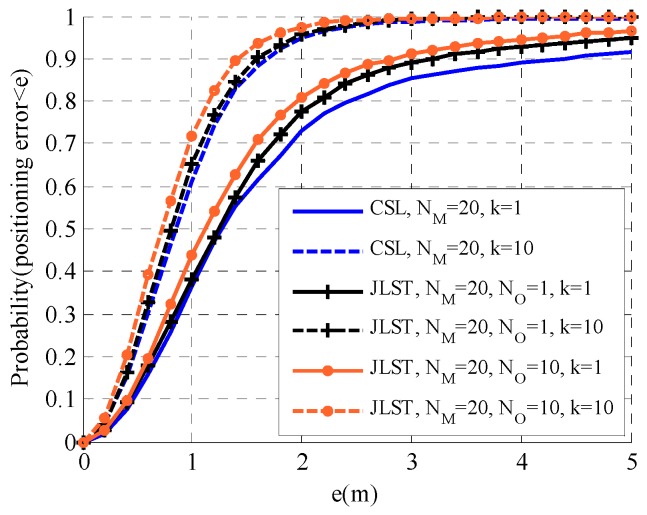
Self-localization performance of the proposed joint self-location tracking JSLT algorithm and the cooperative self-localization (CSL) algorithm ($N_{M}=20$).

**Figure 6:**
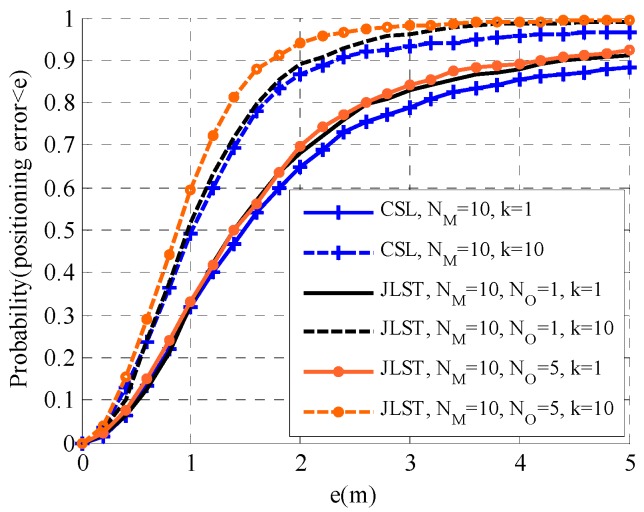
Self-localization performance of the proposed JSLT algorithm and the CSL algorithm ($N_{M}=10$).

**Figure 7:**
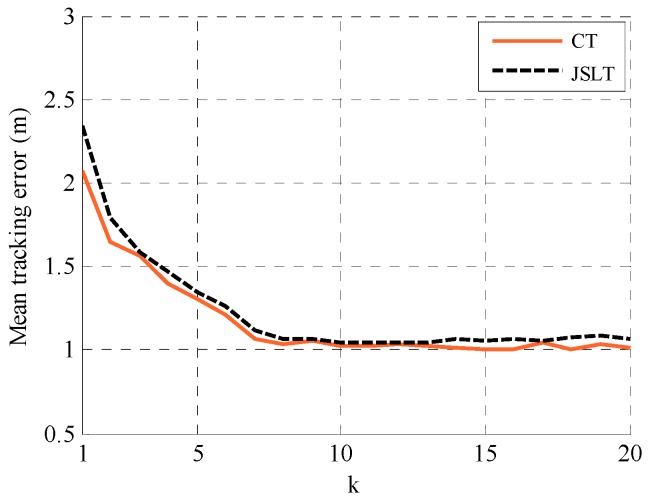
Tracking performance of the proposed JSLT algorithm and the CT algorithm.

The proposed distributed joint self-location and tracking (JSLT) algorithm is evaluated by comparison with the distributed cooperative self-localization algorithm and the centralized target tracking algorithm in this section. We consider a 50 m × 50 m plane with $N_{A}=5$ anchors, $N_{M}$ agents, and $N_{O}$ targets. The communication radius and ranging radius of anchors and agents are $R_{c}=25\;m$, the detection radius is $R_{d}=25\;m$, and the standard variance of ranging measurement noise is 1 m. At the 0th time slot, the variance matrix of the prior of agents and targets are set to be $25I_{2\times 2}$. The iterations of the average consensus algorithm and the joint location algorithm are 20 and 10, respectively. The results in Figure 5, Figure 6 and Figure 7 are obtained by performing 10,000 realizations over 20 time slots.

Self-localization performance of the proposed JSLT algorithm and the cooperative self-localization (CSL) algorithm in [23] with different numbers of agents and targets are shown in Figure 5 and Figure 6. In Figure 5, $N_{M}=20$, $N_{A}=1$ or 10; in Figure 6, $N_{M}=10$, $N_{A}=1$ or 5. It can be seen that the localization performance of both algorithms improves as time increases. This is because the agents’ and their neighbors’ positions become more accurate with the increase in time. However, a higher k value does not always correspond to improved positioning accuracy. The estimated accuracy becomes stable as time goes on. Furthermore, in the two scenarios, the proposed JSLT algorithm has a higher positioning accuracy than the CSL algorithm. This is because, in the JSLT algorithm, agents not only use the distance observation from the neighboring anchors and agents as in the CSL algorithm, but they also make use of the ranging measurements from the targets that they can detect, which improves the self-localization accuracy. Moreover, the performance of the two algorithms is quite different with fewer agents and more targets. When the density of agents is high, agents have enough neighbors. Therefore, they can localize themselves well by cooperating with neighboring anchors and agents. However, when the density of agents is low, many agents have few neighboring anchors and agents to localize themselves. In this case, the ranging measurements from targets play an important role in the self-localization of agents. The computational complexity of both algorithms depends on the number of neighbors. For agent m∈M, we define $N_{m}^{A}$, $N_{m}^{M}$, and $N_{m}^{O}$ to denote the number of neighboring agents, anchors, and targets that can detected at a certain time. The computational complexity of the CSL algorithm and the proposed JSLT algorithm are $O(N_{i}^{A}+N_{i}^{M})$ and $O(N_{i}^{A}+N_{i}^{M}+N_{i}^{O})$, respectively. With regard to communication overhead, as all messages are approximated to Gaussian function, each agent broadcasts the mean and the covariance to its neighbors and collects the information broadcasted by its neighbors. The information collected by each agent is $O(N_{i}^{A}+N_{i}^{M})$ and $O(N_{i}^{A}+N_{i}^{M}+N_{i}^{O})$ for the CSL algorithm and the proposed JSLT algorithm, respectively. Since $N_{i}^{O}\ll N_{i}^{A}+N_{i}^{M}$, the complexity and the communication overhead of the two algorithms are similar.

Figure 7 shows the tracking performance of the proposed JSLT algorithm and the centralized tracking (CT) algorithm is illustrated with $N_{M}=20$ and $N_{A}=1$. In the CT algorithm, agents first localize themselves using the CSL algorithm in [23], then anchors and agents track the targets by sending information to a control center. As shown in Figure 7, the tracking performance of the proposed distributed JSLT algorithm is very close to that of CT algorithm, but the communication overhead of JSLT algorithm is much less than that of CT algorithm. In the CT algorithm, the information collected and broadcasted by each node (anchor or agent) are both proportional to the number of neighbors. In the proposed JLST algorithm, each node broadcasts its global average to its neighbors and collects global averages broadcasted by its neighbors, which is proportional to the number of neighbors. Therefore, the communication overhead sharply reduces by half. Moreover, in the CT algorithm, anchors and agents transmit information to the control center in a multi-hop manner, which leads to a fair amount of redundant information in the networks and information delay.

**Figure 8:**
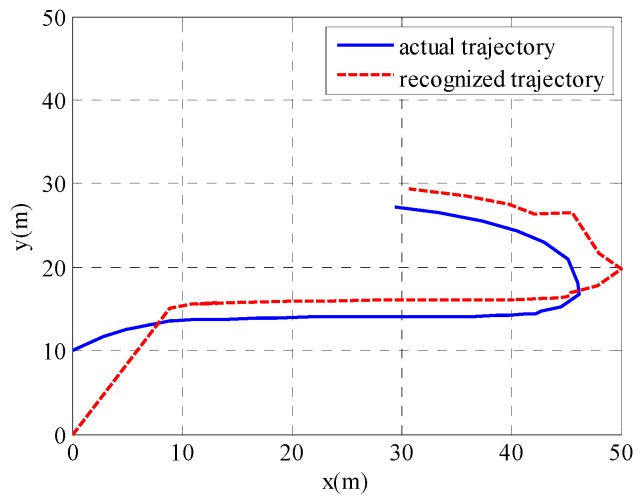
A single trial performance of target tracking.

In order to visualize the tracking performance, a single trial performance of target tracking is shown in Figure 8. We can see that the trajectory recognized by the proposed algorithm is close to the actual trajectory of the target, except for at the initial time and at the moment of a sharp turn.

## 5. Conclusions

In this paper, we built an adaptive prediction model by exploiting the correlation of trajectories and then proposed a joint self-location and tracking algorithm based on VMP and average consensus. Simulation results and analysis illustrated that, compared with instant prediction, linear prediction, and square prediction, the proposed prediction model was more adaptive to the movement of the nodes. The self-location performance of the proposed algorithm is better than the separated cooperative self-localization, and the tracking performance of the proposed algorithm is close to the centralized tracking algorithm with less communication overhead.

The proposed algorithm is suitable for tracking bird migration patterns, monitoring gas diffusion, etc. In these applications, the motion of the targets is random. The following research aimed to apply the proposed approach to practices. In practical networks, delays in data transmission and communication errors are two important issues which affect positioning accuracy and real-time tracking. Future research will focus on the issue of how to deal with the impact of these two issues.

## References

[1] Antolín D., Medrano N., Calvo B. (2016). Reliable Lifespan Evaluation of a Remote Environment Monitoring System Based on Wireless Sensor Networks and Global System for Mobile Communications. J. Sens..

[2] Fakhrulddin S.S., Gharghan S.K., Al-Naji A., Chahl J. (2019). An Advanced First Aid System Based on an Unmanned Aerial Vehicles and a Wireless Body Area Sensor Network for Elderly Persons in Outdoor Environments. Sensors.

[3] Han W., Tian Z., Shi W., Huang Z., Li S. (2019). Low-Power Distributed Data Flow Anomaly-Monitoring Technology for Industrial Internet of Things. Sensors.

[4] Suryadevara N.K., Mukhopadhyay S.C., Kelly S.D.T., Satinder P.S.G. (2015). WSN-Based Smart Sensors and Actuator for Power Management in Intelligent Buildings. IEEE/ASME Trans. Mechatron..

[5] Atzori L., Lera A., Morabito G. (2010). The Internet of Things: A Survey. Comput. Netw..

[6] Fekher K., Abbas B., Abderrahim B., Priyanka R., Mohamed A. (2019). A Survey of Localization Systems in Internet of Things. Mob. Netw. Appl..

[7] Chelouah L., Semchedine F., Bouallouche-Medjkoune L. (2018). Localization Protocols for Mobile Wireless Sensor Networks: A survey. Comput. Electr. Eng..

[8] Taylor C., Rahimi A., Bachrach J., Shrobe H., Grue A. Simultaneous Localization, Calibration, and Tracking in an Ad Hoc Sensor Network. Proceedings of the International Conference on Information Processing in Sensor Networks.

[9] Meyer F., Hlinka O., Wymeersch H., Riegler E., Hlawatsch F. (2016). Distributed Localization and Tracking of Mobile Networks Including Noncooperative Objects. IEEE Trans. Signal Inf. Process. Netw..

[10] Teng J., Snoussi H., Richard C., Zhou R. (2012). Distributed Variational Filtering for Simultaneous Sensor Localization and Target Tracking in Wireless Sensor Networks. IEEE Trans. Veh. Technol..

[11] Djurić P.M., Beaudeau J., Bugallo M.F. Non-Centralized Target Tracking with Mobile Agents. Proceedings of the IEEE International Conference on Acoustics, Speech and Signal Processing, Prague Congress Center.

[12] Xiao L., Boyd S. Fast Linear Iterations for Distributed Averaging. Proceedings of the 42nd IEEE International Conference on Decision and Control (IEEE CDC).

[13] Xiao L., Boyd S., Lall S. A Scheme for Robust Distributed Sensor Fusion Based on Average Consensus. Proceedings of the International Symposium on Information Processing in Sensor Networks.

[14] Olfati-Saber R., Fax J.A., Murray R.M. (2007). Consensus and Cooperation in Networked Multi-Agent Systems. Proc. IEEE.

[15] Farahmand S., Roumeliotis S.I., Giannakis G.B. (2011). Set-Membership Constrained Particle Filter: Distributed Adaptation for Sensor Networks. IEEE Trans. Signal Process..

[16] Hlinka O., Hlawatsch F., Djuric P.M. (2014). Consensus-based Distributed Particle Filtering with Distributed Proposal Adaptation. IEEE Trans. Signal Process..

[17] Gu D., Sun J., Hu Z., Li H. Consensus Based Distributed Particle Filter in Sensor Networks. Proceedings of the International Conference on Information and Automation.

[18] Ghirmai T. Distributed Particle Filter Using Gaussian Approximated Likelihood Function. Proceedings of the 48th Conference on Information Sciences and Systems.

[19] Hlinka O., Sluciak O., Hlawatsch F., Djuric P.M., Rupp M. (2012). Likelihood Consensus and Its Application to Distributed Particle Filtering. IEEE Trans. Signal Process..

[20] Wymeersch H., Lien J., Win M.Z. (2009). Cooperative Localization in Wireless Networks. Proc. IEEE.

[21] Yuan W.J., Wu N., Etzlinger B., Wang H., Kuang J. (2016). Cooperative Joint Localization and Clock Synchronization Based on Gaussian Message Passing in Asynchronous Wireless Networks. IEEE Trans. Veh. Technol..

[22] Cui J., Wang Z., Zhang C., Sun P., Zhu Z. (2016). Variational Message Passing-based Localisation Algorithm with Taylor Expansion for Wireless Sensor Networks. IET Commun..

[23] Cui J., Wang Z., Zhang C., Zhang Y., Zhu Z. (2017). Message Passing Localisation Algorithm Combining BP with VMP for Mobile Wireless Sensor Networks. IET Commun..

[24] Camp T., Boleng J., Davies V. (2002). A Survey of Mobility Models for Ad Hoc Network Research. Wirel. Commun. Mob. Comput..

